# Mapping the cellular and molecular heterogeneity of normal and malignant breast tissues and cultured cell lines

**DOI:** 10.1186/bcr2755

**Published:** 2010-10-21

**Authors:** Patricia J Keller, Amy F Lin, Lisa M Arendt, Ina Klebba, Ainsley D Jones, Jenny A Rudnick, Theresa A DiMeo, Hannah Gilmore, Douglas M Jefferson, Roger A Graham, Stephen P Naber, Stuart Schnitt, Charlotte Kuperwasser

**Affiliations:** 1 Department of Anatomy & Cellular Biology, Sackler School, Tufts University School of Medicine, 136 Harrison Ave, Boston, MA 02111, USA; 2 Molecular Oncology Research Institute, Tufts Medical Center, 75 Kneeland St, Boston, MA 02111, USA; 3 Department of Pathology, Beth Israel Deaconess Medical Center, Harvard Medical School, 330 Brookline Avenue, Boston, MA 02215, USA; 4 Department of Physiology, Sackler School, Tufts University School of Medicine, 136 Harrison Ave, Boston, MA 02111, USA; 5 Department of Surgery, Tufts Medical Center, 750 Washington St., Boston, MA 02111, USA; 6 Department of Pathology, Tufts Medical Center, 750 Washington St., Boston, MA 02111, USA

## Abstract

**Introduction:**

Normal and neoplastic breast tissues are comprised of heterogeneous populations of epithelial cells exhibiting various degrees of maturation and differentiation. While cultured cell lines have been derived from both normal and malignant tissues, it remains unclear to what extent they retain similar levels of differentiation and heterogeneity as that found within breast tissues.

**Methods:**

We used 12 reduction mammoplasty tissues, 15 primary breast cancer tissues, and 20 human breast epithelial cell lines (16 cancer lines, 4 normal lines) to perform flow cytometry for CD44, CD24, epithelial cell adhesion molecule (EpCAM), and CD49f expression, as well as immunohistochemistry, and *in vivo *tumor xenograft formation studies to extensively analyze the molecular and cellular characteristics of breast epithelial cell lineages.

**Results:**

Human breast tissues contain four distinguishable epithelial differentiation states (two luminal phenotypes and two basal phenotypes) that differ on the basis of CD24, EpCAM and CD49f expression. Primary human breast cancer tissues also contain these four cellular states, but in altered proportions compared to normal tissues. In contrast, cultured cancer cell lines are enriched for rare basal and mesenchymal epithelial phenotypes, which are normally present in small numbers within human tissues. Similarly, cultured normal human mammary epithelial cell lines are enriched for rare basal and mesenchymal phenotypes that represent a minor fraction of cells within reduction mammoplasty tissues. Furthermore, although normal human mammary epithelial cell lines exhibit features of bi-potent progenitor cells they are unable to differentiate into mature luminal breast epithelial cells under standard culture conditions.

**Conclusions:**

As a group breast cancer cell lines represent the heterogeneity of human breast tumors, but individually they exhibit increased lineage-restricted profiles that fall short of truly representing the intratumoral heterogeneity of individual breast tumors. Additionally, normal human mammary epithelial cell lines fail to retain much of the cellular diversity found in human breast tissues and are enriched for differentiation states that are a minority in breast tissues, although they do exhibit features of bi-potent basal progenitor cells. These findings suggest that collections of cell lines representing multiple cell types can be used to model the cellular heterogeneity of tissues.

## Introduction

Human breast cell lines have long served as models for a wide array of applications including the study of molecular, cellular, and biochemical mechanisms that regulate breast epithelial biology. Breast cancer cell lines are also commonly used in xenograft models for drug discovery and in the assessment of pre-clinical experimental therapeutic efficacy. Despite their crucial role for rational drug discovery and development and in understanding molecular pathophysiology of cancer, their ability to accurately reflect phenotypes of tumors remains controversial. Several studies have suggested that cell lines exhibit a narrow range of genetic profiles, harbor genetic alterations due to adaptation of tissue culture environment, and are poor predictors of *in vivo *sensitivity to drug efficacy [1-3]. Cell line-derived xenograft models also fail to recapitulate the heterogeneous histopathology characteristic of the parent tumor histology. However, other studies have indicated that cell lines, as a system, actually mirror many of the biological and genomic properties found within primary human tumors [4,5]. Genomic approaches have revealed that like primary tumors, the gene expression signatures of breast cancer cell lines can distinguish luminal from basal subtypes of breast cancer [6-9]. Moreover, cell line-derived gene signatures can correctly classify human tumor samples [6,7,10], suggesting that despite their acquired ability to grow *in vitro*, and acquired mutations following adaptation to culture conditions, cell lines continue to share many of the molecular and genetic features of the primary breast cancers from which they were derived.

The use of primary human breast tissues for experimental studies and breast cancer research has been fueled by the notion that cell lines are not accurate models of the heterogeneity found *in vivo*. As such, reduction mammoplasty and cancer tissues have been used to identify and characterize epithelial differentiation states and lineages since it is presumed that not all cell types are maintained or mirrored *in vitro*. Expression of epithelial cell adhesion molecule (EpCAM) and CD49f^+ ^(α6 integrin) have been used to identify luminal and basal/myoepithelial cells from breast tissues [11-14]. Mature luminal cells are reported to express an EpCAM^+^/CD49f^- ^phenotype while luminal progenitors express an EpCAM^+^/CD49f^+ ^marker profile. Myoepithelial cells and basal progenitor cells are defined by an EpCAM^-^/CD49f^+ ^phenotype [11,13,15]. In addition to EpCAM and CD49f, surface expression of CD44 and CD24 have also been used to identify luminal epithelial cells that express genes involved in hormone responses (CD24^+^) and cells resembling progenitor cells that express genes involved in motility (CD44^+^) [16].

Reflecting the normal cell types within the breast, tumors are broadly classified histopathologically by expression of either luminal cytokeratins (CK8/18) or stratified epithelial cytokeratins (CK5/6/14, basal-type) [17,18]. Similarly, tumor subclasses identified by microarray were named to reflect the gene expression patterns of the normal breast luminal and myoepithelial/basal cells [19-23]. Luminal-type breast cancers (Luminal A and Luminal B) express estrogen receptor (ER). Her2-type breast cancers typically overexpress or amplify Her2, are generally negative for ER expression and tend to express the genes associated with the Her2-amplicon. Lastly, Basal-like breast cancers are also often referred to as triple-negative tumors since they do not express ER, progesterone receptor (PR), or Her2 [19-22].

To determine if cell lines mirror or maintain the cellular differentiation states found in primary tissues, we examined the molecular and cellular profiles of normal and malignant human breast epithelial cell lines and compared them to normal and cancerous tissues. In doing so, we found four distinguishable cell states across a collection of cell lines that mirrored the four differentiation states present within normal and malignant breast tissues. However, we also found that the cellular heterogeneity within cell lines was remarkably restricted in culture and was enriched for cellular phenotypes that were normally present as a minor component *in vivo*.

## Materials and methods

### Cell lines and tissue culture

SUM cell lines were obtained from Dr. Stephen Ethier (Kramanos Institute, Detroit, MI, USA) and are commercially available (Asterand, Detroit, MI, USA). The MCF7, T47 D, BT20, MCF10A, MCF10F, MDA.MB.231, MDA.MB.361 and HCC cell lines were obtained directly from the American Type Culture Collection (ATCC; Manassas, VA, USA). The MCF10A and MCF10F cell lines are non-tumorigenic mammary epithelial cell lines that were produced by long-term culture in serum-free medium with low calcium; the MCF10A cells were derived from an the adherent population in these cultures, while the MCF10F line was derived from floating cells within the MCF10 cultures [24]. All of the ATCC cell lines used in this study were low passage (< 10). SUM225CWR, SUM149PT, and SUM159PT cells were cultured in F12 with 5% calf serum (CS), insulin (5 μg/ml), and hydrocortisone (1 μg/ml), while SUM1315 MO2 cells were cultured in F12 with 5% CS, insulin (5 μg/ml), and hEGF (10 ng/ml). MCF7, MDA.MB.361, BT20, and all HCC cell lines were cultured in DMEM with 10% fetal bovine serum (FBS; Invitrogen, Carlsbad, CA, USA). MDA.MB.231 and T47 D cells were cultured in Roswell Park Memorial Institute-1640 (RPMI; Hyclone, Logan, UT, USA) with 10% FBS. The TUM177 breast cancer cell line was established from a primary invasive ER-positive adenocarcinoma. An ER-negative cancer cell line spontaneously emerged after two months of cultivations. TUM177 cells were cultured in DMEM with 10% fetal bovine serum (FBS; Invitrogen, Carlsbad, CA, USA).

HME I and HME II cells were derived from reduction mammoplasty tissues from two different patients grown in Mammary Epithelial Growth Medium (MEGM) until the generation of variant cells [25] and then immortalized through the ectopic expression of the catalytic subunit of human telomerase (hTERT) [26].

MCF10F cells were cultured in Dulbecco's modified Eagle's medium-Ham's F12 (DMEM/F12; 1:1) with 5% horse serum, insulin (5 μg/ml), hydrocortisone (1 μg/ml), and human epidermal growth factor (hEGF; 10 ng/ml), and cholera toxin (100 ng/ml) (all, Sigma, St. Louis, MO, USA). MCF10A and immortalized human mammary epithelial (HME) cell lines were cultured in MEGM supplemented with bovine pituitary extract (52 μg/ml), hydrocortisone (0.5 μg/ml), hEGF (10 ng/ml) and insulin (5 μg/ml) (MEGM Bullet Kit, Lonza Corporation, Walkersville, MD, USA). MCF10A cells were further supplemented with cholera toxin (100 ng/ml). For serum differentiation experiments, HME or MCF10A cells were switched to growth in the MCF10F medium with substitution of 5% CS for the horse serum and omission of the cholera toxin, or 5% CS was added to MEGM and cells were allowed to differentiate for six days before use in experiments. For mammosphere culture, cells were plated at 20,000 cells/ml and grown on ultra-low adherence six-well plates for one week (Corning Life Sciences, Lowell, MA, USA). Quantification of mammospheres was accomplished using a Multisizer 3 COULTER COUNTER (Beckman-Coulter, Brea, CA USA) that provides number, and size distributions with an overall sizing range of 14 μm to 336 μm.

### Reduction mammoplasty and tumor tissue specimens

All human breast tissue procurement for these experiments was obtained in compliance with the laws and institutional guidelines, as approved by the Institutional Review Board committee from Beth Israel Deaconess Hospital and Tufts Medical Center. Fresh disease-free reduction mammoplasty tissues (n = 12) and tumor tissues (n = 15; 8 fresh, 15 formalin-fixed paraffin embedded) were obtained from discarded material from patients undergoing elective reduction mammoplasty surgeries or from patients undergoing partial or complete mastectomy for excision of tumor tissue from the Pathology departments at BIDMC or Tufts Medical Center. All samples were obtained from de-identified discarded material and therefore, informed consent was not required for these studies. All samples were evaluated histologcially and confirmed to be invasive ductal carcinomas. The following histopathologic variables, determined for all tumor tissue specimens, were done on full sections, and cases with 10% or more positive for ER, p53 or EGFR staining were grouped as positive. The scoring of Her2 was performed using the ASCO/CAP guidelines, as follows: Cases with 30% or more strongly positive cells with strong complete membrane staining were defined as Her2+ tumors. Cases with 10% or more positive cells with weak to moderate complete membrane staining were considered Her2+ but were not defined as Her2+ tumors solely on this basis. IHC analysis for estrogen receptor (ER), progesterone receptor (PR), Her2, p53 and EGFR were independently reviewed by expert breast pathologists (HG and SN). Breast tumor subtypes were defined as follows: Luminal A (ER+ and/or PR+, Her2-), Luminal B (ER+ and/or PR+, Her2+), Her2+ (ER-, PR-, Her2+), and Basal-like (ER-, PR-, Her2-, and epidermal growth factor receptor (EGFR)+/-) and p53+.

Uncultured cells from reduction mammoplasty or human breast tumor organoid preps [27] were dissociated to a single-cell suspension by trypsinization and filtered through a 20 μm nylon mesh (Millipore, Danvers, MA, USA). Human breast tumors were plated in DMEM supplemented with 10% CS for one to two hours to deplete stromal cells.

### Immunohistochemical analysis and scoring

Immunohistochemistry was performed by the Histology Special Procedures Laboratory at Tufts Medical Center on paraffin-embedded tissue sections on a Ventana (Tucson, Arizona, USA) automated slide stainer with the iVIEW DAB detection kit for visualization. Antibodies used were CK14 (1:500, clone LL002, Vector (Burlingame, CA, USA)), CK8/18 1:500, clone DC-10, Vector), Vimentin (1:500, clone V9, Vector), S100A4 (1:200, clone 1F12-1G7, Sigma), S100A6 (1:200, clone CACY-100, Sigma), p53 (Ventana Medical Systems), ER (Ventana Medical Systems), Her2 (Ventana Medical Systems), EGFR (1:20, clone 31G7, Zymed), and PR (Ventana Medical Systems). All Ventana antibodies are prediluted.

IHC and IF results were semi-quantitatively analyzed in a blinded fashion across multiple patient samples using a scoring metric in 10% increments. Negative staining represents 0 to 10% of the cell staining and was given a score of 1; mixed staining represents moderate to strong intensity staining of cells with > 10% but < 50% positive cells and was given a score of 2; and positive staining represents strong intense staining with > 50% cells staining positive and was given a score of 3. The staining intensity and percent staining scores were added to obtain a total stain score for each field. An average total stain score was calculated for the staining for a particular sample. Statistical analysis was performed using the student's t-test across the different patient samples.

### Flow cytometry and FACS

Uncultured cells from reduction mammoplasty tissues (n = 12) or primary breast tumor tissues (n = 8) from organoid preparations were dissociated to single-cell suspensions, as described above. For reduction mammoplasty tissues, endothelial, lymphocytic, monocytic, and fibroblastic lineages were depleted with antibodies to CD31, CD34 and CD45 (all Thermo/LabVision, Fremont, CA, USA) and Fibroblast Specific Protein/IB10 (Sigma) using a cocktail of Pan-mouse IgG and IgM Dynabeads (Dynal, Invitrogen) according to the manufacturers instructions and as described previously [28]. Depleted single cells suspensions were resuspended at 1 × 10^6 ^cells/ml in phosphate-buffered saline containing 1% calf serum (FACS buffer, FB) and bound with fluorescently-conjugated antibodies to human EpCAM (APC), CD49f (PE), and CD24 (FITC) (all, BD Biosciences, San Jose, CA, USA) for 20 minutes at 4°C. Antibody-bound cells were washed and resuspended at 1 × 10^6 ^cells/ml in FB and run on a FACSCalibur flow cytometer. Flow cytometry data was analyzed with the Flowjo software package (TreeStar, Ashland, OR, USA).

For fluorescence-activated cell sorting (FACS), cells from reduction mammoplasty tissue were prepared as above for flow cytometry and resuspended at 5 x10^6 ^cells/ml in FB and sorted on a BD Influx Cell sorter (BD Biosciences) into culture medium (MEGM) containing 50% CS.

For cell lines, non-confluent cultures of cells were trypsinized into single cell suspension, counted, washed with PBS, and stained with antibodies specific for human cell CD24 (PE) and CD44 (APC) (BD Biosciences). The cells were stained with antibodies specific for human cell surface markers: EpCAM-fluorescein isothiocyanate (FITC), CD24-phycoerythrin (PE), and CD49f-PE-Cy5 or CD44-allophycocyanin (APC) (BD Biosciences). Additional cells were stained with isotype controls for each antibody: Ms IgG_1_-FITC, Ms IgG_2a_-PE, and Rat IgG_2a_-PE-Cy5 or Ms IgG_2b_-APC (BD Biosciences). A total of 200,000 to 800,000 cells were incubated with antibodies or isotype controls for 20 minutes on ice. The cells were washed with PBS to remove any unbound antibody and analyzed no later than one hour post-staining on a FACSCalibur flow cytometer (BD Biosciences). Antibody-bound cells were resuspended at 1 × 10^6 ^cells/ml in FB and run on a FACSCalibur flow cytometer (BD Biosciences) or sorted on an BD Influx FACS sorter (BD Biosciences). Flow cytometry data was analyzed with the Flowjo software package (TreeStar). Each cell line was analyzed in three to five different biological replicates.

### Immunofluorescence

Collected cell fractions from FACS were counted and cytospun onto glass slides at 10,000 cells per spot with a Cytospin 4 cytospinner (Thermo Scientific, Waltham, MA, USA). Cultured cell lines were plated at 10 to 20,000 cells per well in eight-well chamber slides (BD Biosciences) and grown two to three days. Cytospins and cells in chamber slides were fixed in 100% methanol and stained overnight at 4°C with primary antibodies directed to EpCAM (VU-ID9, 1:100, Stem Cell Technologies, Vancouver, BC, Canada), CK8/18 (5D3, 1:500, Vector Labs, Burlingame, CA, USA), ERα (1D5, 1:100, Santa Cruz Biotechnology, Santa Cruz, CA, USA) CK14 (ASM-1, 1:500, Thermo Scientific/LabVision), α-smooth muscle actin (SMA; 1:250, Vector Labs) and vimentin (V9, 1:500, Vector Labs) followed by secondary antibodies (1:500 Alexa488 or Alexa555 conjugated anti-mouse and anti-rabbit H+L IgG, Invitrogen) for one hour at room temperature. Nuclei were counterstained with 4', 6-diamidino-2-phenylindole (DAPI) and images were captured with the Spot imaging software (Diagnostic Instruments, Inc., Sterling Heights, MI, USA); staining was analyzed by counting the total number of cells positive stain compared to the total number of cells in multiple fields with at least 50 cells analyzed per condition. Negative staining represents no cells staining positive, Mixed staining is > 1% but < 50% of the cells staining positive, while positive staning is > 80% of the cells staining positive.

An average total stain score of a cell line was calculated using three to five different regions of the plate. Statistical analysis was performed using the student's T-test across the different patient samples.

### Animals and surgery

All animal procedures were performed in accordance with an approved protocol submitted to the Tufts University Institutional Animal Care and Use Committee. A colony of NOD/SCID mice was maintained under sterile conditions and received food and water *ad libum*. Nulliparous female mice aged 8 to 10 weeks were utilized in all experiments. For tumor latency studies, 1 × 10^6 ^human breast cancer cells were resuspended in media and Matrigel (1:1; BD Biosciences) and injected orthotopically in a total of 4 to 10 different glands. Tumor formation was assessed by palpitation at least once a week, and tumor growth curves were calculated from weekly caliper measurements as previously described. Tumor latency is described as the time it takes for a tumor to reach a diameter of 1 cm.

### Statistical analysis

Fisher exact tests were used when comparing the binary categories of expression of proteins between groups. All *P-*values reported are two-sided.

## Results

### CD44 and CD24 expression in human breast cancer cell lines

Studies have suggested that the pre-existing differentiation state of normal precursor cell types is so strongly encoded it survives the neoplastic transformation and accounts in part for tumor phenotype [29,30]. Based on this notion, we reasoned that it might be possible to map different tumor subtypes to their normal cellular precursors within human breast tissues based on the expression of cell surface markers. Recently, the cell surface markers CD24 and CD44 have been used to define normal human breast epithelial differentiation states: CD44 is expressed in basal cells while CD24 is expressed in luminal cells [16].

**Figure 1:**
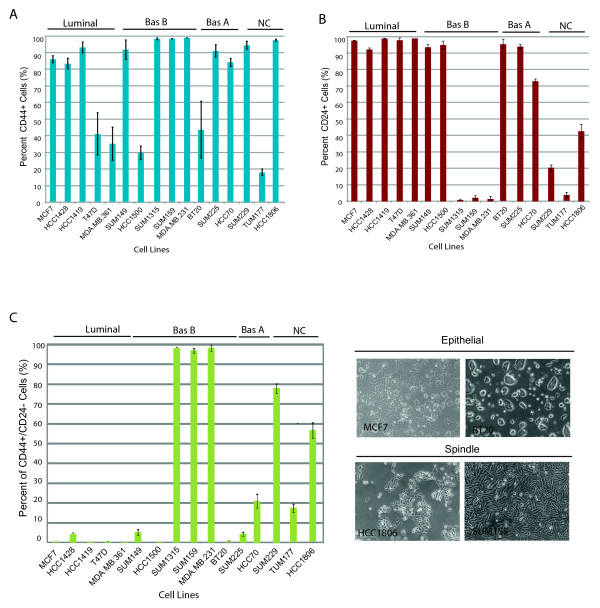
**CD44 or CD24 expression alone does not classify breast cancer cell lines into tumor subytpes**. Breast cancer cell lines are grouped based on tumor subtype classification defined by [7] as Luminal, Basal A (Bas A), Basal B (Bas B), or those that have not been previously classified (NC). The percentage of cells in breast cancer cell lines expressing either CD44 **(a) **or CD24 **(b) **is variable and does not correlate with tumor classification. **(c) **The percentage of cells in cancer cell lines expressing the CD44^+^/CD24^- ^phenotype correlates with spindle/mesenchymal features, not tumor subtype. Surface marker expression was quantified by flow cytometry (mean ± S.E.) as described in Materials and methods. Phase contrast, bright-field photomicrographs of representative cell lines exhibiting epithelial versus spindle morphology.

We wanted to determine whether these markers could be used to classify luminal and basal breast cancer cell lines, many of which have been previously classified on the basis of gene expression profiling [7,31]. Using a panel of 16 cancer lines we found that all breast cancer cell lines contained a population of CD44^+ ^cells regardless of tumor subtype. Most of the lines (11/16) contained a majority (> 80%) of CD44^+ ^cells, while the remaining cell lines (5/16) contained a minority (< 40%) of CD44^+ ^cells (Figure 1a, Additional files 1 and 2). There was no correlation (*P *= 0.14, *P *= 0.44, *P *= 1) between the proportion of CD44^+ ^(greater than or 80% or less than 40%) cells within the cell line with breast cancer subtype.

In contrast to CD44 expression, not all breast cancer cell lines contained CD24^+ ^cells. Rather, 10/16 lines contained a large proportion (> 70%) of CD24^+ ^cells, while 6/16 lines contained very few (< 5 to 45%) CD24^+ ^cells (Figure 1b, Additional files 1 and 2). As with CD44 expression, there was no correlation between the proportion of CD24^+ ^cells in cell lines and tumor subtype. Since CD44 and CD24 expression alone could not be used to classify cell lines based on tumor subtype, we examined whether together these markers might be able to categorize cell lines. While, the proportion of CD44^+^/CD24^- ^cells did not correlate with gene expression-based classifiers of breast cancer subtype, consistent with previous reports, there was a striking relationship between the proportion of CD44^+^/CD24^- ^cells in the line and spindle-cell morphology (Figure 1c), [32,33] (Additional files 1, 2, 3 and 4).

### EpCAM, CD24 and CD49 expression reduction mammoplasty tissues

Since CD44 and CD24 were not useful markers to classify tumor cells, we wanted to determine whether additional lineage markers might be able to refine cellular differentiation states. Accordingly, we used flow cytometry to characterize breast epithelial cells from reduction mammoplasty tissues (n = 12) using EpCAM, and CD49f expression. EpCAM and CD49f have been used previously to define cells within the luminal and basal lineages from normal human breast tissue [11,14,15].

**Figure 2:**
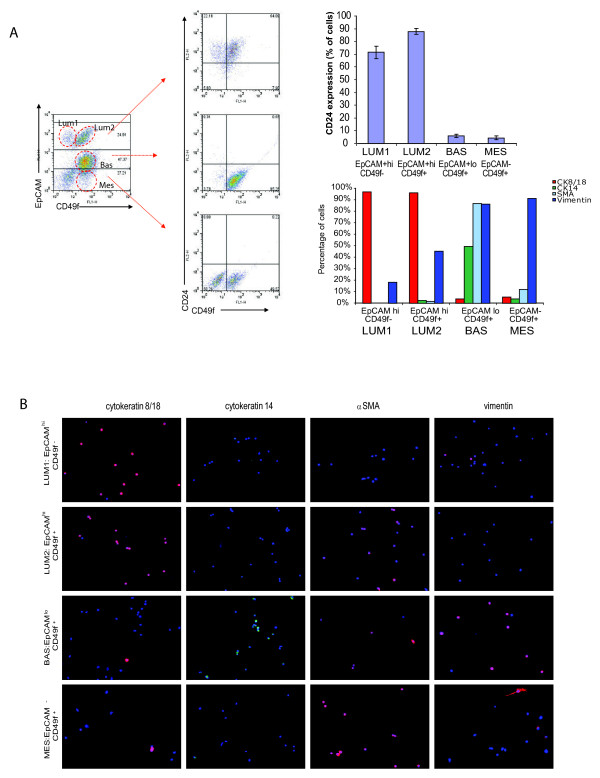
**Normal human breast tissue contains four distinct epithelial subtypes**. **(a) **Freshly dissociated breast epithelial cells from reduction mammoplasty can be divided into four epithelial differentiation states. Primary breast epithelial cells (n = 12) were isolated, lineage depleted, stained with EpCAM, CD24, and CD49f, and quantified as described in Materials and methods. Representative dot plots of EpCAM vs. CD49f staining (left) and CD24 vs. CD49f staining in EpCAM/CD49f populations (middle) are shown. Quantification of CD24 staining in Luminal 1/2, Basal and Mesenchymal populations from 12 patient samples (right, mean ± S.E.). Quantification of immunofluorescence from a representative patient sample is shown (% of total DAPI stained cells, minimum 50 cells analyzed). Luminal 1 and Luminal 2 cells from reduction mammoplasty tissue are predominantly CK 8/18 positive. Basal and Mesenchymal cells expressed CK14, VIM, and αSMA. **(b) **Freshly dissociated epithelial cells from reduction mammoplasty tissue were stained for EpCAM and CD49f expression, sorted by FACS and cytospun on onto slides for characterization of lineage markers by immunofluorescence. Cytokeratin (CK) 18, 14, smooth muscle actin (αSMA) and vimentin (VIM) immunofluorescence staining and quantification of sorted populations indicates luminal and basal/myoepithelial cell enrichment.

We identified four epithelial cell populations (two populations of luminal cells and two populations of basal cells) from freshly dissociated, lineage-depleted breast epithelial cells from reduction mammoplasty tissues on the basis of EpCAM/CD24/CD49f expression (Figure 2). There were three populations of cells identified on the basis of EpCAM expression; EpCAM^hi ^cells, which expressed CD24 but were either CD49f^+ ^or CD49f^-^, EpCAM^low ^cells that lacked CD24 expression but expressed CD49f, and EpCAM-negative cells that also lacked CD24 expression but were CD49f-positive.

To confirm the nature of these cell types, we sorted lineage-depleted cells from reduction mammoplasty tissues by FACS, and cytospun freshly sorted cells to examine the expression of established markers of luminal and myoepithelial/basal cells (Figure 2b, c). Highly expressing EpCAM^+ ^luminal cells were either CD49f^+ ^or CD49f^-^, consistent with the definition of mature luminal cells and luminal progenitor cells, respectively [14,15]. EpCAM^+^/CD49f^- ^and EpCAM^+/^CD49f^+ ^cells were predominantly CK8/18 positive, lacked CK14 and SMA expression thus were termed Luminal 1 and Luminal 2 cells, respectively. EpCAM^+^/CD49f^- ^and EpCAM^+^/CD49f^+ ^cells also both expressed CD24. However, unlike previous reports, we observed a second EpCAM^+^/CD49f^+ ^population of cells that expressed lower levels of EpCAM. Unlike EpCAM^+^/CD49f^+ ^luminal progenitor cells, this population of EpCAM^+^/CD49f^+ ^cells lacked CD24 expression. In addition, EpCAM^+^/CD24^-^/CD49f^+ ^cells were predominantly CK14-positive, while EpCAM^+^/CD24^+^/CD49f^+ ^cells were predominantly CK18-postive (Figure 2). Furthermore, EpCAM^+^/CD24^-^/CD49f^+ ^cells expressed SMA and vimentin; and thus were termed Basal. Finally, an EpCAM-negative population which lacked CD24 expression was also identified. This population expressed CD49f, expressed lower levels of CK14, and strong levels of vimentin (Figure 2b, c). Although EpCAM^-^/CD49f^+ ^cells expressed basal epithelial markers, they were termed mesenchymal, due to the lack of luminal epithelial markers (CD24 and EpCAM), and the higher levels of vimentin expression.

### Cellular and molecular heterogeneity in breast cancer tissues

**Figure 3:**
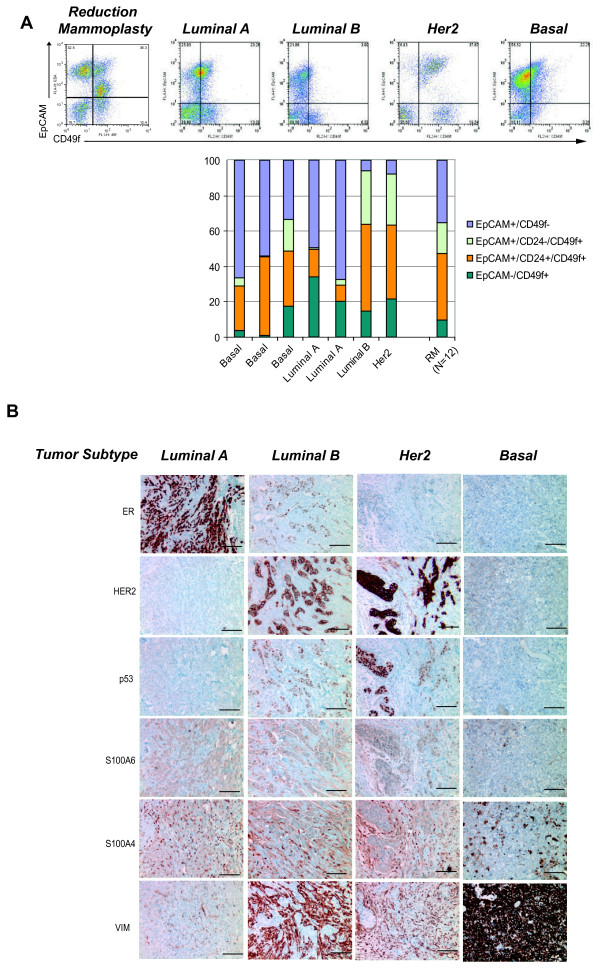
**Human breast cancers are heterogeneous tissues comprised of all four cell phenotypes**. Primary breast tumors exhibited a similar cellular heterogeneity to normal reduction mammoplasty tissues when examined by flow cytometry using the markers EpCAM, CD24, and CD49f as described in Materials and methods. **(a) **Representative flow cytometry dot plots of EpCAM and CD49f expression in reduction mammoplasty tissues (RM) and primary human breast tumors of different subtypes. Quantification of flow cytometry tumors (n = 8) classified clinically by expression of ER, PR and Her2 expression. **(b)** Primary breast cancers demonstrated heterogeneous expression of markers used to characterize cancer cell line xenografts by immunohistochemistry as in Figure 2. Bar = 100 μm.

To determine whether these four epithelial cell types were also present within breast cancer tissues, we analyzed freshly dissociated breast epithelial cells from primary human breast cancers (n = 8) by flow cytometry. Primary tumor tissues, in general, showed a different spectrum of cellular heterogeneity compared to breast reduction mammoplasty tissue by flow cytometry when stained for EpCAM, CD49f, and CD24 (Figure 3a). Although the four major cell types were still present regardless of the tumor classification (Luminal (A or B), Her2, Basal), several tumor tissues contained a larger proportion of EpCAM^-^/CD49f^+ ^Mesenchymal cells compared to reduction mammoplasty tissues. Although the number of tumors analyzed was too small to make any statistically significant conclusions, it was interesting to note that basal tumors, which have been considered to express mesenchymal markers, contained the fewest numbers of EpCAM^-^/CD49f^+ ^Mesenchymal cells, while Her2-positive tumors, which are traditionally viewed as a subset of luminal tumors, retained the fewest numbers of EpCAM^+^/CD49f^- ^Luminal 1 cells. It will be interesting to determine if these observations can be expanded across a wider spectrum of tumor specimens.

We also analyzed breast cancer tissues (n = 15) by immunohistochemistry for markers of Luminal 1, Luminal 2, Basal and Mesenchymal cells. Consistent with the flow cytometry data, human breast cancers exhibited heterogeneous and variable expression of markers of Luminal 1, Luminal 2, Basal and Mesenchymal cells, regardless of tumor subtype (Figure 3b). Future prospective studies are needed to determine whether the differences in cell state proportions within tumors are associated with clinical and prognostic information.

### EpCAM, CD24 and CD49 epithelial subtypes in breast cancer cell lines

**Figure 4:**
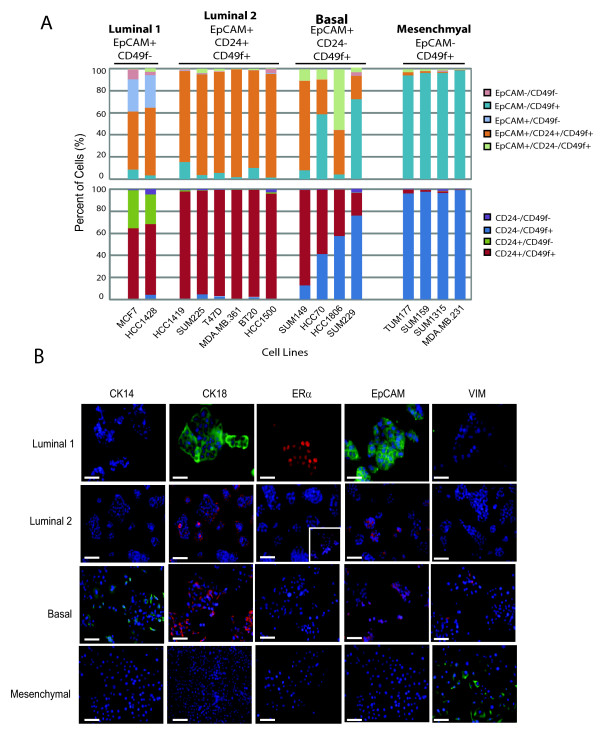
**Surface markers EpCAM, CD24, and CD49f classify breast cancer cell lines into distinct differentiation states**. **(a) **Cell surface markers EpCAM, CD24, and CD49f distinguish four classes of cell lines that map to differentiation states found in normal precursor cells. Surface marker expression was quantified by flow cytometry (mean ± S.D., n = 3 to 4 biological replicates per cell line) as described in Materials and Methods. **(b) **Luminal 1, Luminal 2, Basal, and Mesenchymal cell lines identified by EpCAM, CD24, and CD49f expression were classified on the basis of CK14, CK8/18, ERα, EpCAM, and vimentin expression. Representative immunofluorescence images are shown from MCF7 (Luminal 1), BT20 (Luminal 2), HCC1806 (Basal), and MDA.MB.231 (Mesenchymal). Nuclei were counterstained with DAPI (blue). ER expression of Luminal II cells is heterogeneous across cell lines. Cell lines T47 D, HCC1500, and MDA.MB.361 all express ER (MDA.MB.361 is shown in inset), while BT20, SUM225 and HCC1419 are ER-negative. Original magnification: 200×. Bar = 100 μm.

All 16 breast cancer cell lines were analyzed for the expression of EpCAM, CD24, and CD49f to determine whether the same four differentiation states present within human breast tissues were retained in cultured lines. While we indeed observed the presence of all four of these differentiation states within the panel of human breast cancer cell lines, the majority of cell lines failed to retain Luminal 1 EpCAM^+^/CD49f^- ^cells. Rather only one class of cell lines could be readily distinguished from all other lines by retaining this population of EpCAM^+^/CD24^+^/CD49f^- ^cells (Figure 4a, Additional files 4, 5 and 6); these cell lines are thereafter referred to as Luminal 1-type lines. Luminal 1 cell lines were derived from pleural effusions, and are strongly ER-positive, thus of the luminal subtype. A second class of cell lines were distinguished by a prominent population (> 90%) of EpCAM^+^/CD24^+^/CD49f^+ ^luminal cells and are thus referred to as Luminal 2 lines (Figure 4a, Additional files 4 and 5). Luminal 2 cell lines (6/16) included cell lines that were derived from either pleural effusions or primary tumor tissues and express ER, Her2 or both ER and Her2 (Figure 4a, Additional files 4 and 6). The third class of cell lines could be distinguished by two prominent populations (> 15%) of EpCAM^+^/CD49f^+ ^cells: EpCAM^+^/CD24^+^/CD49f^+ ^luminal cells and EpCAM^+^/CD24^-^/CD49f^+ ^basal cells, the latter of which were rare or absent in other cell lines. Thus, these cancer lines were referred to as Basal lines (Figure 4a, Additional files 4 and 6). All Basal cell lines (4/16) in this category were derived from primary breast tumors and are ER-, PR-, and Her2-negative. Finally, cell lines that exhibited a spindle-like morphology in culture, were derived from either pleural effusions or primary tumor tissues and were largely comprised of EpCAM^-^/CD24^-^/CD49f^+ ^Mesenchymal cells (> 90%) (Figure 4a, Additional files 4 and 6); thus, referred to as Mesenchymal lines. Notably, all Mesenchymal cell lines lack ER, PR and Her2 expression.

Consistent with previous reports, we observed a strong association between the cell surface-based categories, morphology and molecular markers. Luminal cells (Luminal 1 and 2) grew as epithelial-differentiated monolayers with tight cell-cell junctions. They all expressed CK8/18 and EpCAM, and all lacked expression of the basal cytokeratin CK14 and mesenchymal vimentin (Figure 4b, Additional files 4 and 6). In contrast, Mesenchymal cells appeared less differentiated and exhibited a spindle-like appearance. They lacked expression of both of CK8/18 and CK14 expression and were all strongly positive for vimentin expression (Figure 4b, Additional files 4 and 6). Interestingly, Basal cell lines generally exhibited a more scattered morphology compared to Luminal cell lines but were more epithelial compared to Mesenchymal cell lines. Consistent with their luminal-like morphology, Basal cell lines all expressed CK8/18 and EpCAM, but they all also expressed the basal maker CK14 (Figure 4b, Additional files 4 and 6), which was absent in both Luminal and Mesenchymal cell lines. Moreover, vimentin expression was rarely detected in Basal lines and when it was, it was focal and restricted to rare cells within the population (Additional files 4 and 6). These findings indicate that breast cancer cell lines retain the four cell differentiation states that map to normal precursors found in reduction mammoplasty tissues.

### *In vivo *tumorigenicity and growth characteristics of human breast cancer cell lines

**Figure 5:**
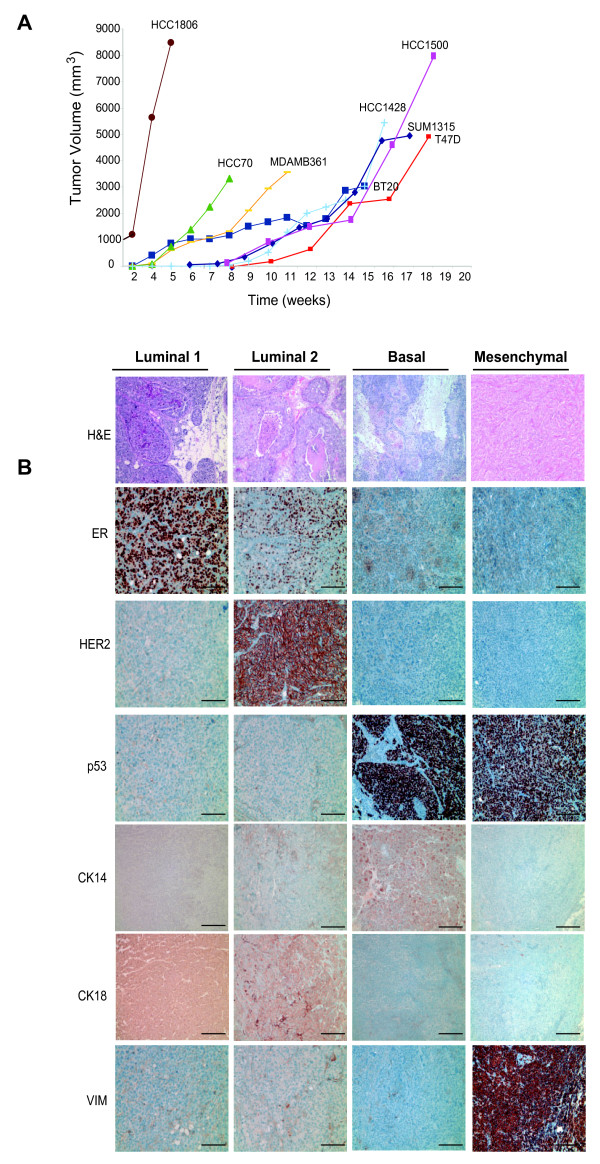
**Cell line marker profiles correlate with established biomarkers in tumor xenografts**. **(a) **Tumor growth curves of cell line-derived xenografts over time. Tumorigenicity of breast cancer cell lines was not correlated with their individual cellular profiles when injected into mammary glands of NOD/SCID mice. **(b) **Cell line marker profile correlated with resulting tumor histology and expression of biomarkers. Representative H&E stained sections of tumor xenografts from Luminal 1 and 2 cell lines (MCF7, SUM225) showing intraductal and comedo-like DCIS patterns of growth respectively, as well as, Basal cell line (HCC1806) showing solid carcinoma growth with squamous differentiation, and Mesenchymal cell lines (SUM159) showing spindle-cell metaplastic-like growth. Original magnification: 100×. Immunohistochemistry was used to stain tumor xenografts for expression of ERα, Her2, p53, CK14, CK18 and vimentin. Representative images shown from tumors of Luminal 1 (MCF7), Luminal 2 (MDA.MB.361), Basal (SUM 149), and Mesenchymal (MDA.MB.231) cell lines. Bar = 100 μm.

We injected all 16 breast cancer cell lines into immunodeficient NOD/SCID mice and assessed each line for tumor formation, invasiveness and histopathology of the xenografts (Figure 5). Xenograft tumors that developed from adherent cancer cell lines were all poorly differentiated, high grade carcinomas. Despite the lack of differentiation, the cell line definition did correlate with morphologic features and the expression of established biomarkers within the tumors (Figure 5b, Additional file 6). Luminal 1, Luminal 2, and Basal cancer cell lines all formed solid epithelial carcinomas in mice, some of which exhibited both invasive and *in situ *ductal or comedo-like growth patterns, or squamous differentiation features. In contrast, Mesenchymal cell lines formed solid carcinomas that lacked obvious ductal features and exhibited metaplastic and/or carcino-sarcoma differentiation (Figure 5b, Additional files 6 and 7). Luminal 1 cell lines formed tumors that were exclusively ER-positive and negative for p53, vimentin and Her2. Luminal 2 cell lines also formed tumors that expressed either ER and/or Her2, but failed to express p53 or vimentin (Figure 5b, Additional files 6 and 7). Basal cell lines formed tumors that expressed robust p53 but lacked ER and Her2 expression (Figure 5b, Additional files 6 and 7). Basal tumors also lacked vimentin expression with the exception of the tumor-stromal interface (data not shown). Unlike Luminal and Basal cell lines, Mesenchymal cancer cell lines formed almost exclusively spindle-cell metaplastic tumors that lacked obvious epithelial features (Figure 5b, Additional files 6 and 7). In addition, tumors derived from Mesenchymal lines were strongly and uniformly positive for vimentin and p53, consistent with clinical basal-like tumors (Figure 4b, Additional files 6 and 7). However, unlike primary human basal-like breast cancers that have been reported to express EGFR protein, EGFR expression in cell-line derived xenograft tumors was only weakly expressed in HCC1806 and TUM177 xenografts and not expressed preferentially in tumors derived from other Basal or Mesenchymal cell lines despite its expression in these cultured cell lines (Additional files 6 and 7, and [10]).

### Enrichment for basal phenotypes in normal breast cell lines

**Figure 6:**
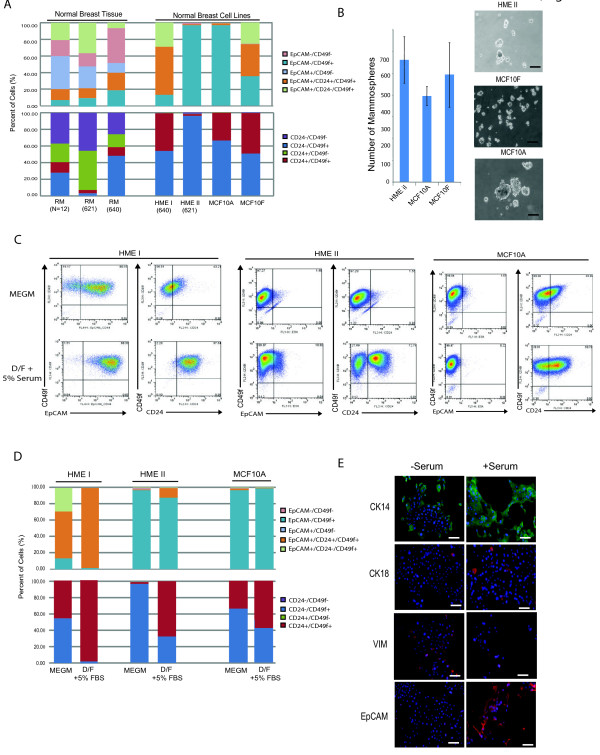
**Human breast cell lines are enriched for basal and mesenchymal phenotypes**. **(a) **Normal breast cell lines demonstrate loss of EpCAM^+^/CD49f- and EpCAM^+^/CD24^+^/CD49f^+ ^populations compared to primary breast epithelial cells isolated from reduction mammoplasty. Reduction mammoplasty tissues (RM) and normal breast cells lines as well as matched RM with HME cell lines were stained with EpCAM, CD24 and CD49f and quantified by flow cytometry as described in Materials and methods. **(b) **Quantification of mammospheres formed in non-adherent culture by HME II, MCF10A and MCF10F cell lines (left) and representative images (right). Bar = 100 μm. **(c, d) **Addition of 5% serum to the culture conditions of HME cells increases differentiation to a more luminal state as assessed by flow cytometry for EpCAM, CD49f and CD24. Representative dot plots are shown in C and quantification in D. **(e) **Changes in Basal/Luminal differentiation were assessed by immunofluorescence staining for CK8/18, EpCAM, CK14 and vimentin following treatment with serum. Cells were counterstained with DAPI (blue). HME I cells are shown, bar = 100 μm.

Since the majority of breast cancer cell lines failed to maintain EpCAM^+^/CD24^+^/CD49f^- ^Luminal 1 cells *in vitro*, we wanted to determine whether this was a general feature of *in vitro *cell cultivation or was a consequence of malignancy. We therefore compared non-transformed human breast epithelial cell lines (HMECs (HME I, HME II), MCF10A and MCF10F) with reduction mammoplasty tissues for cell surface and molecular features. Surprisingly, we found that under serum-free conditions none of the normal human mammary epithelial cell lines contained Luminal 1 cells in culture, nor could they be classified as Luminal 2 cells. Rather normal human breast epithelial cell lines were classified into two categories: Basal lines (HME I and MCF10F cell lines) that contained a prominent Basal population, and Mesenchymal lines (HME II and MCF10A cell lines) that were comprised of a majority (> 90%) Mesenchymal EpCAM^-^/CD24^-^/CD49f^+ ^cells (Figure 6a). These data indicate that the selection for basal and mesenchymal cell states in cultured breast epithelial cells is not a consequence of genetic mutation or malignant transformation, but is likely the result of adherent *in vitro *selection.

We used immunofluorescence to determine whether non-transformed Basal and Mesenchymal cell lines expressed similar markers of normal reduction mammoplasty counterparts (Figure 6e). In contrast to Mesenchymal cancer cell lines, which failed to express CK8/18 or CK14 and grew as spindle cells, normal Mesenchymal epithelial cell lines expressed both CK14 and vimentin, and grew as cobblestone islands of cells, suggesting they retained some of the molecular features of normal Mesenchymal epithelial cells found in reduction mammoplasty tissues. In addition, Basal mammary cell lines expressed CK8/18 and CK14 but also expressed vimentin, reminiscent of Basal cells in breast tissues. These data suggest that normal Basal and Mesenchymal cell lines may retain more features that mirror differentiation in reduction mammoplasty tissues than Basal and Mesenchymal cells in cancer cell lines.

The expression of CK14, CK8/18, and vimentin combined with the high CD44 expression in HMEC cultures (data not shown) suggested that Basal and Mesenchymal cells may retain characteristics of bi-potent progenitor cells. Mammosphere formation is associated with the ability to generate cells of both breast lineages in culture [34]. Therefore, we performed mammosphere assays to gauge progenitor activity in normal mammary epithelial cell lines. Indeed, HME I, HME II, MCF10A and MCF10F cells all formed mammospheres at similar rates, although MCF10A cells formed much larger spheres compared to the other lines (Figure 6b, data not shown). The potential progenitor activity of HMEC cultures combined with the obvious absence of EpCAM^+^/CD24^+^/CD49f^- ^Luminal 1 cells prompted us to determine whether Basal or Mesenchymal lines could differentiate and give rise to Luminal 1 cells *in vitro*. It has been reported that luminal-type cells are growth-promoted in the presence of serum while basal/mesenchymal cells are selected for in the presence of serum-free media, which is the typical growth medium for HMECs [35]. Therefore, we treated HME I/II and MCF10A cell lines with serum and assessed whether this might affect the differentiation of cells into Luminal 1 cells. The addition of serum to Basal HME I cells indeed led to the development of a Luminal 2 cell line due to an increase in the proportion of EpCAM^+^/CD24^+^/CD49f^+ ^cells (> 90%) and the loss of EpCAM^+^/CD24^-^/CD49f^+ ^cells (Figure 6c, d). However, the addition of serum failed to induce differentiation of Luminal 1 cells. In contrast to Basal lines, the addition of serum to Mesenchymal lines only resulted in a modest increase in Luminal 2 cells. However, a significant increase in the proportion of CD24^+ ^luminal cells lacking EpCAM expression was observed in Mesenchymal cell lines. Since this cell type does not exist in any significant proportion in reduction mammoplasty tissues, it is unclear what type of luminal cell this is.

The expansion of Luminal 2 cells was confirmed by immunofluorescence for expression of lineage markers CK8/18 expression and EpCAM expression (Figure 6e). Collectively, these results indicate that *in vitro *cultivation of human breast epithelial cells selects for the Mesenchymal and Basal cells which retain the capacity to differentiate into EpCAM^+^/CD24^+^/CD49f^+ ^Luminal 2 cells or CD24^+ ^cells.

## Discussion

We have used flow cytometry and immunostaining for lineage markers to identify four epithelial cell states present within normal human breast epithelial tissues and have shown that these cell states can be used to stratify a panel of human breast cancer cell lines. Through use of a three-marker strategy, we have subdivided human breast tissue into Luminal 1 cells, characterized by the majority of cells having an EpCAM^hi^CD24^+^CD49f^- ^profile; Luminal 2 cells, characterized by a majority of EpCAM^hi^CD24^+^CD49f^+ ^cells; Basal cells, characterized by EpCAM^+/lo^CD24^-^CD49f^+ ^cells, and Mesenchymal cells, characterized by EpCAM^-^CD24^-^CD49f^+ ^cells. Our description of four major cell types within breast tissue is similar to previously published reports describing epithelial populations through the use of EpCAM and CD49f staining [11-15]. Notably, Villadsen *et al*. described two luminal populations representing lobular and ductal-oriented luminal cells characterized as EpCAM^hi^CD49f^- ^and EpCAM^hi^CD49f^+^, respectively, and lobular and ductal myoepithelial/basal populations with EpCAM^lo/-^CD49f^+ ^phenotypes [11].

Recently, several groups have identified breast bi-potent progenitor/stem-like activity in EpCAM^+/hi^CD49f^+ ^populations but also in EpCAM^-/lo^CD49f^+ ^populations [11-15]. These conflicting differences may arise from use of different fluorescently conjugated antibodies for flow cytometry and gating strategies. Alternatively, it could be that human breast tissue may contain two distinct populations of bi-potent stem/progenitor cells. Consistent with this notion, ductal (CD24^lo^CD49f^hi^) and lobular/alveolar (CD24^hi^CD49f^lo^) progenitors that both give rise to luminal and myoepithelial cells have been described in the mouse mammary gland [36,37]. By using CD24 to further define luminal populations in human breast tissues, it may be that EpCAM^hi/+^/CD24^-^/CD49f^+ ^and EpCAM^lo/+^/CD24^-^/CD49f^+ ^represent the lobule and ductal progenitors in the human breast. CD24^+ ^cells have been previously described to be associated with the EpCAM^+^CD49f^+ ^luminal progenitors [14]. However, we have observed that CD24^+ ^cells are found in both the EpCAM^hi^CD49f^- ^and EpCAM^hi^CD49f^+ ^populations. It is worth speculating that the use of CD24 as an additional marker might reveal different bi-potent potentials of progenitor cells. Indeed, we found that HMEC lines with bi-potent and differentiation potential contained EpCAM^+^/CD24^-^/CD49f^+ ^cells, while those that were nearly all EpCAM^-^/CD49f^+ ^cells were only able to differentiate into an EpCAM^-^/CD24^+ ^phenotype which does not exists in human breast tissue. Therefore, future studies that further define the normal breast epithelial cell hierarchy using additional markers will be necessary to fully understand the complex cell types and differentiation states in human tissues.

In this small study, we surprisingly found that the majority of human breast cancer tissues exhibited a EpCAM^+^/CD49f^+ ^luminal epithelial differentiation phenotype regardless of their molecular subtype. This is consistent with immunohistochemistry studies that have reported that breast cancers largely express luminal makers despite being of the basal molecular subtype [38]. We found that in tissues and cell lines, the EpCAM^+^/CD49f^+ ^phenotype contains both CD24^+ ^and CD24^- ^cells. In reduction mammoplasty tissues, EpCAM^+^/CD24^-^/CD49f^+ ^cells exhibited a basal cytokeratin phenotype while breast cancer cell lines with a basal-like phenotype also contained a unique population of EpCAM^+^/CD24^-^/CD49f^+ ^cells. Gene expression profiling of cell lines that exhibit a large EpCAM^+^/CD49f^+ ^population most closely corresponded with the expression profile of Basal-like breast tumors [14] suggesting that EpCAM^+^/CD49f^+ ^cells may be the cellular precursors to both luminal and basal-like tumors. Future studies will need to be performed to determine if this is indeed the case.

We found that adherent cultures of normal human breast epithelial cells and to a lesser extent, cancer cell lines lead to enrichment of cells that exhibited basal and mesenchymal differentiation states with limited capacity to differentiate into fully-committed luminal cells. This suggests that standard adherent culture may select preferentially for cells of basal-orientation, or may result in epigentic loss of luminal differentiation programs.

Data from studies in mouse mammary glands and human tissues suggest that bi-potent progenitor/stem-like activity is correlated with the formation of colonies that contain cells of both luminal and basal lineages, defined by keratin CK8/18/19 or CK14/5 expression, respectively. However, since luminal cells are lost following *in vitro *cultivation, this suggests that bi-potent progenitor/stem-like activity from luminal cells has not been well studied. This does not discount the evidence that mammary stem-like cells have basal characteristics but it does suggest that *in vitro *methods need to be improved to allow for maintenance or cultivation of cells of the luminal lineage to better model cells that are likely of great importance for human breast tumor development.

In this study, we found that the morphology and molecular classification of several cell lines differed from those previously reported by others [7,40,41]. In this study, all the commercially available cell lines were obtained directly from ATCC or from Dr. Ethier, were characterized at low passage (less than 10 passages) and were grown in specified medium. Under these conditions, we found a strong association between epithelial or spindle-cell morphology, marker expression (CK14, CK18, vimentin, and EpCAM), and the proportion of CD44^+^/CD24^- ^cells. It is well established that cancer cell lines evolve over time in culture and may be influenced by a variety of factors including confluency, media compositions as well as passage number. Thus, it is highly likely that as certain cell lines have evolved in culture when grown under differing conditions and in turn have acquired different morphological features. However, it is likely the case that such cell lines could still be classified on the basis of cell surface phenotypes and be grouped into one of the four breast epithelial differentiation states. Future studies will be needed to determine whether the plasticity of the cell state dynamics within cancer cell lines is due to *de novo *acquired mutations or due to epigenetic changes associated with extracellular environment.

## Conclusions

Our data indicate that, while cell lines as a group indeed represent the heterogeneity of human breast tumors, individually, they exhibit a notable increase in lineage-restricted profiles that falls short of truly representing the intratumoral heterogeneity of individual breast tumors, regardless of their molecular classification. This is in large part due to the loss of Luminal 1 cells in culture, which represents a major cell phenotype of normal and malignant breast tissues. Additionally, we found that normal human breast epithelial cell lines, like cancer cell lines, have a Basal/Mesenchymal-restricted lineage phenotype under normal serum-free culture conditions but that they can be induced to partially differentiate under serum-containing conditions. However, the four normal breast cell lines tested, representing some of the most commonly used cell lines for studying the behavior of mammary epithelial cells in culture, have a phenotype that does not represent the major cell types within breast tissue, namely, differentiated luminal epithelial cells and luminally-oriented progenitors. These results serve as a resource for further understanding the behavior and origins of breast cell lines, which are crucial and widely used research models. However, they also demonstrate that additional models and cell lines are needed to more accurately depict and study human breast epithelial cell types and tumors in a manner that is more efficient for developing effective therapies. These findings also indicate that further studies are needed to identify culture conditions that can allow for the growth and expansion of Luminal 1 cells, which seem to be unable to survive or expand *in vitro*.

## Abbreviations

APC: allophycocyanin; CK: cytokeratin; CS: calf serum; DAPI: 4',6-diamidino-2-phenylindole; DCIS: ductal carcinoma *in situ*; DMEM: Dulbecco's modified Eagle's medium; EMT: epithelial to mesenchymal transition; EpCAM: epithelial cell adhesion molecule; ER: estrogen receptor; F12: Ham's F12 medium; FACS: fluorescence activated cell sorting; FB: flow buffer; FBS: fetal bovine serum; FITC: fluorescein isothiocyanate; hEGF: human epidermal growth factor; HME: human mammary epithelial; HMEC: human breast epithelial cell; hTERT: human telomerase; MEBM: mammary epithelial basal medium; MEGM: mammary epithelial growth medium; PBS: phosphate buffered saline; PE: phycoerythrin; PR: progesterone receptor; RPMI: Roswell Park Memorial Institute-1640 medium; SMA: smooth muscle actin.

## Competing interests

The authors declare that they have no competing interests.

## Authors' contributions

PJK, AL, LMA and ADJ took part in the conception and design of the study, the collection of data, data analysis and interpretation, and manuscript writing. IK collected and/or assembled data. CF and CMP took part in the collection of data, and data analysis and interpretation. JAR and TAD collected data. HG, SS, RAG, DJ and SN dealt with the provision of study materials, including the procurement of resources and samples (cell lines or reduction mammoplasty and tumor tissues). CK took part in the conception and design of the study, the collection and/or assembly of data, data analysis and interpretation, manuscript writing and financial support. All authors approved of the final manuscript.

## Supplementary Material

Additional file 1 **Morphology and surface markers EpCAM, CD24, and CD49f classify breast cancer cell lines into distinct differentiation states**. Human Luminal breast cancer cell lines can be classified into Luminal 1 or Luminal 2 cell lines based on morphology in tissue culture (left panels, original magnification: 100×) and by expression of EpCAM, CD24 and CD49f cell surface markers (dot plots, right panels). Cell lines were stained for EpCAM, CD24, and CD49f and quantified by flow cytometry as described in Materials and methods. Click here for file

Additional file 2 **Morphology and surface markers EpCAM, CD24, and CD49f classify breast cancer cell lines into distinct differentiation states**. Human Basal breast cancer cell lines can be classified into Basal or Mesenchymal cell lines based on morphology in tissue culture (left panels, original magnification: 100×) and by expression of EpCAM, CD24 and CD49f cell surface markers (dot plots, right panels). Cell lines were stained for EpCAM, CD24, and CD49f and quantified by flow cytometry as described in Materials and Methods. Click here for file

Additional file 3 **CD44^+^/CD24^-^/EpCAM^+ ^cells are variable across a panel of cultured human breast cell lines**. Human breast cancer cell lines were stained for EpCAM, CD24, and CD44 and quantified by flow cytometry as described in Materials and Methods. Cell staining CD44^+^/CD24^- ^(upper left quadrant, dot plots) were analyzed for the percentage of EpCAM^+ ^cells, which is shown in the histogram to the right of the dot plots. The percentage of CD44^+^/CD24^-^/EpCAM^+ ^cells is calculated by multiplying the percentage of EpCAM^+ ^cells by the percentage of CD44^+^/CD24^+ ^cells. Click here for file

Additional file 4 **Luminal 1, Luminal 2, Basal, and Mesenchymal cell lines identified by EpCAM, CD24, and CD49f expression were classified on the basis of CK14, CK8/18, ERα, EpCAM, and vimentin expression**. Representative immunofluorescent images are from the panel of Luminal 1, Luminal 2, Basal, and Mesenchymal cell lines. Nuclei were counterstained with DAPI (blue). Original magnification: 200×. Click here for file

Additional file 5 **Table 1**. Molecular and cellular characterization of human breast cell lines. Click here for file

Additional file 6 **Table 2**. Histopathological characteristics of breast cancer cell line xenografts. Click here for file

Additional file 7 **Table 3**. *In vitro *vs. *in vivo *comparative molecular marker expression of breast cancer cell lines. Click here for file
