# Supplementary figures and images for: Mapping the cellular and molecular heterogeneity of normal and malignant breast tissues and cultured cell lines
Source: Breast Cancer Res. 2010 Oct 21;12(5):R87. doi: 10.1186/bcr2755 (PMC3096980; doi:10.1186/bcr2755)

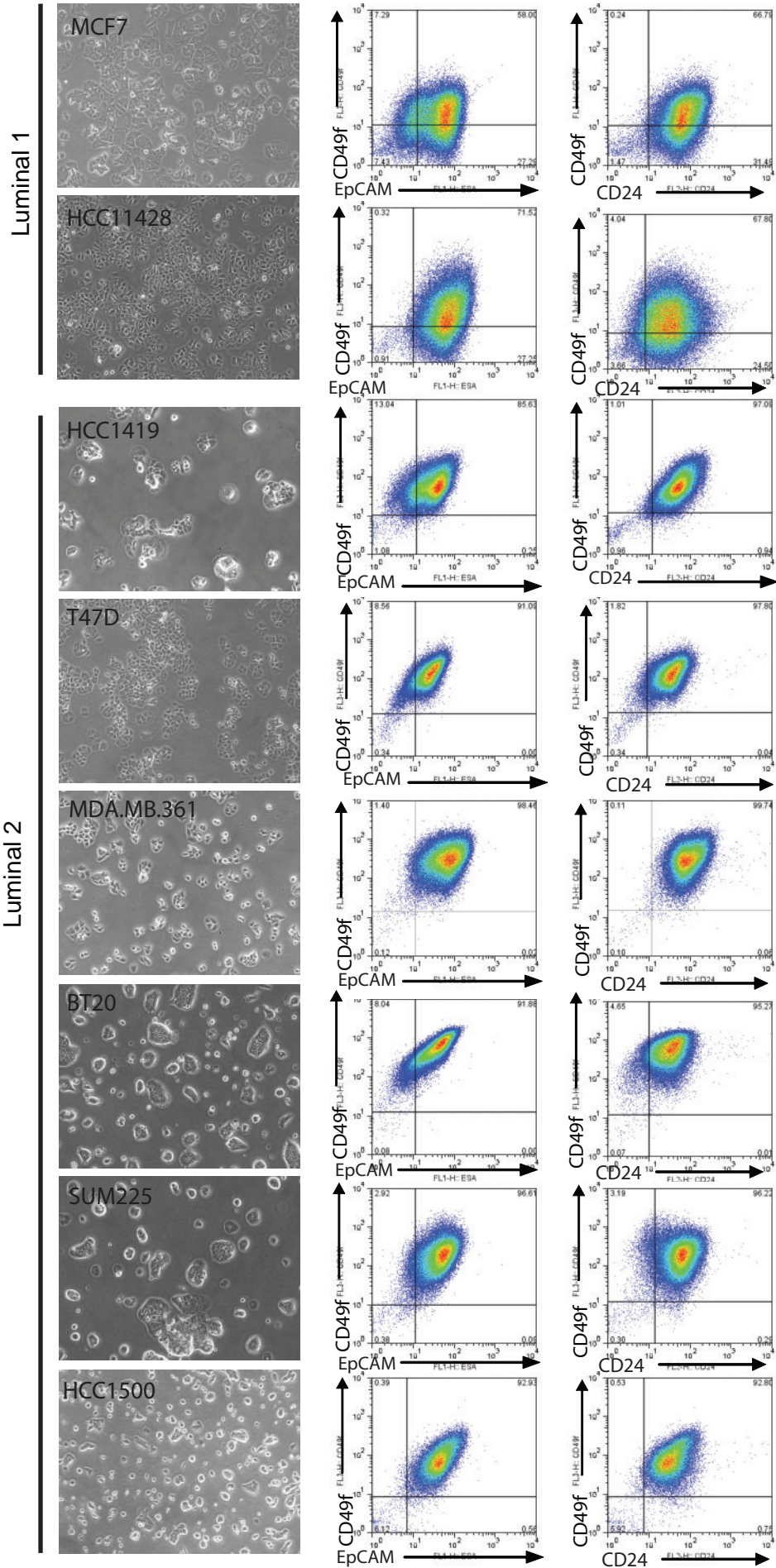

Supplement: Additional file 1 — Morphology and surface markers EpCAM, CD24, and CD49f classify breast cancer cell lines into distinct differentiation states. Human Luminal breast cancer cell lines can be classified into Luminal 1 or Luminal 2 cell lines based on morphology in tissue culture (left panels, original magnification: 100×) and by expression of EpCAM, CD24 and CD49f cell surface markers (dot plots, right panels). Cell lines were stained for EpCAM, CD24, and CD49f and quantified by flow cytometry as described in Materials and methods. [file bcr2755-S1.PDF]

Basal

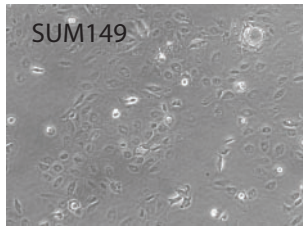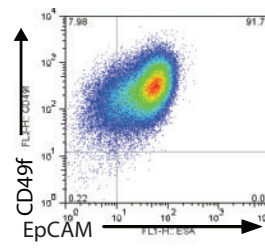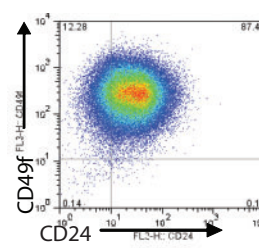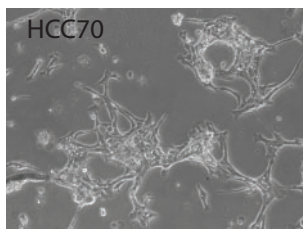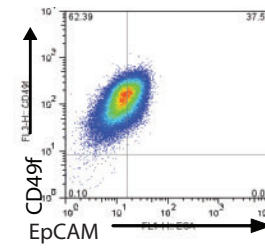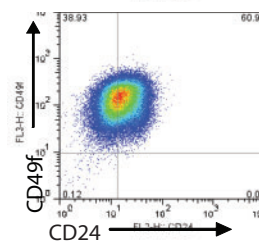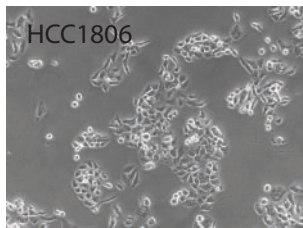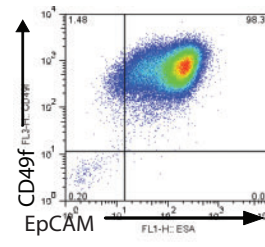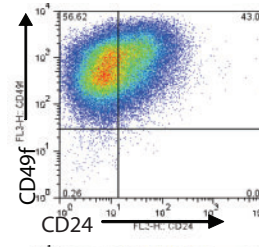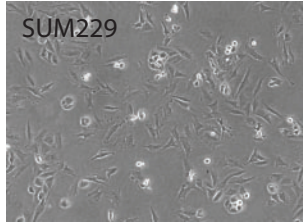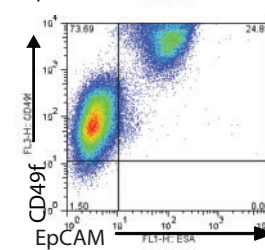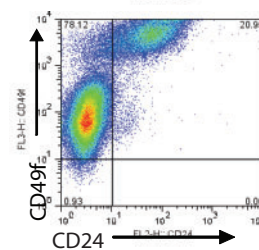

Mesenchymal

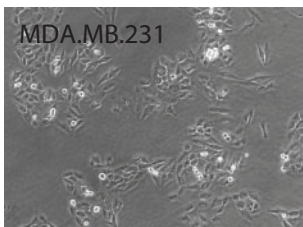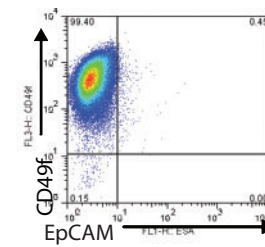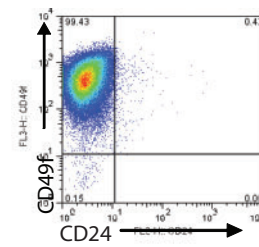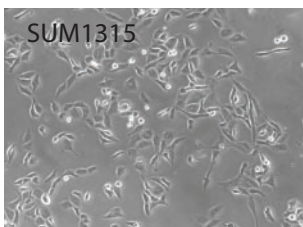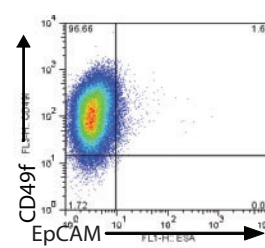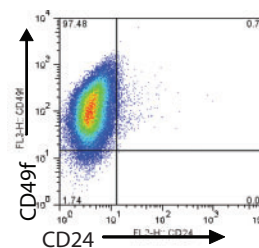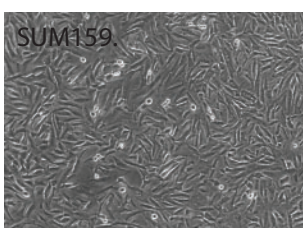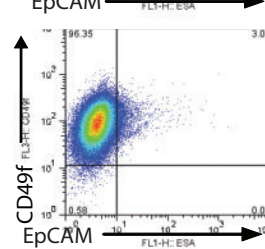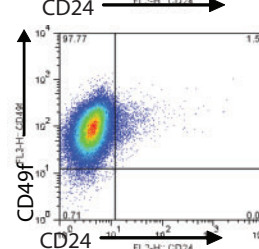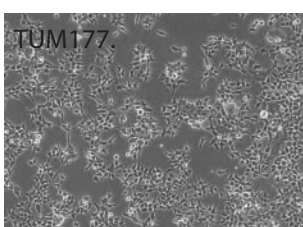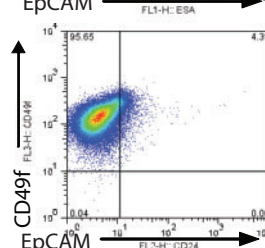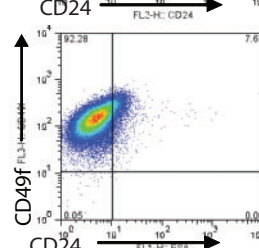

Supplement: Additional file 2 — Morphology and surface markers EpCAM, CD24, and CD49f classify breast cancer cell lines into distinct differentiation states. Human Basal breast cancer cell lines can be classified into Basal or Mesenchymal cell lines based on morphology in tissue culture (left panels, original magnification: 100×) and by expression of EpCAM, CD24 and CD49f cell surface markers (dot plots, right panels). Cell lines were stained for EpCAM, CD24, and CD49f and quantified by flow cytometry as described in Materials and Methods. [file bcr2755-S2.PDF]

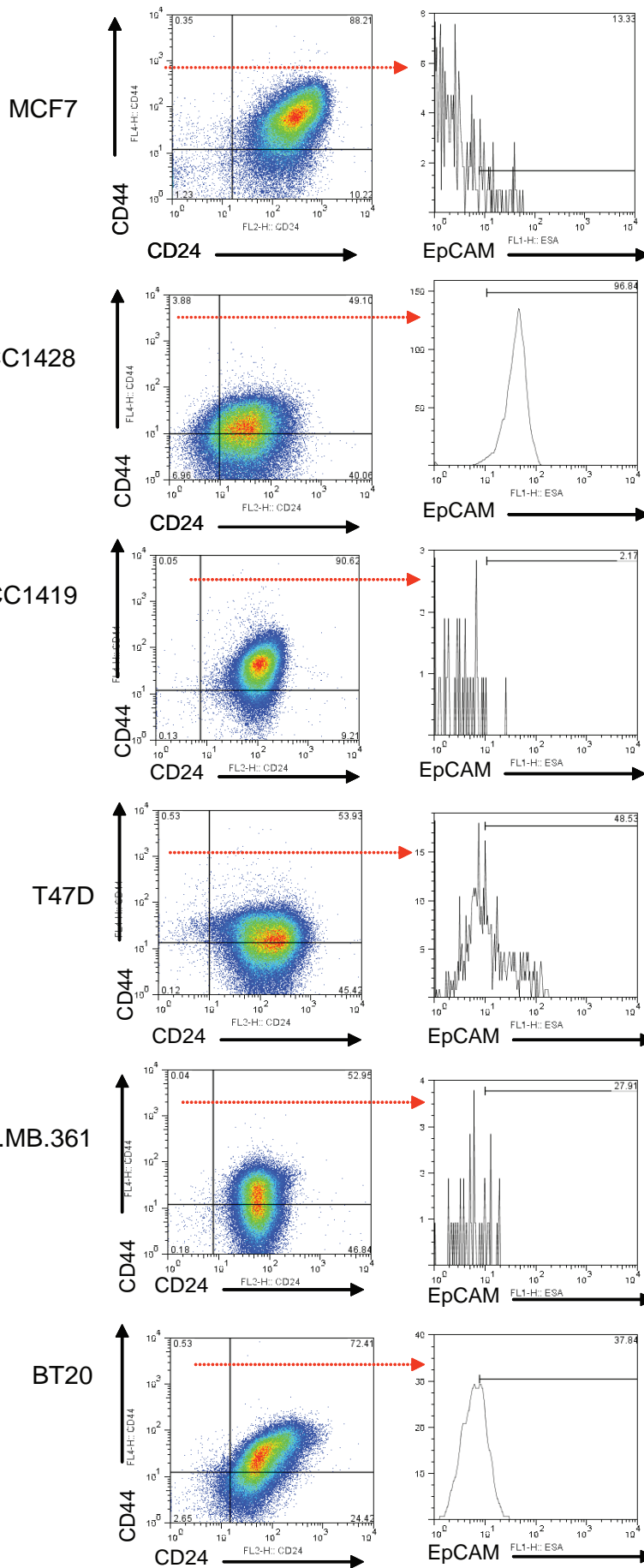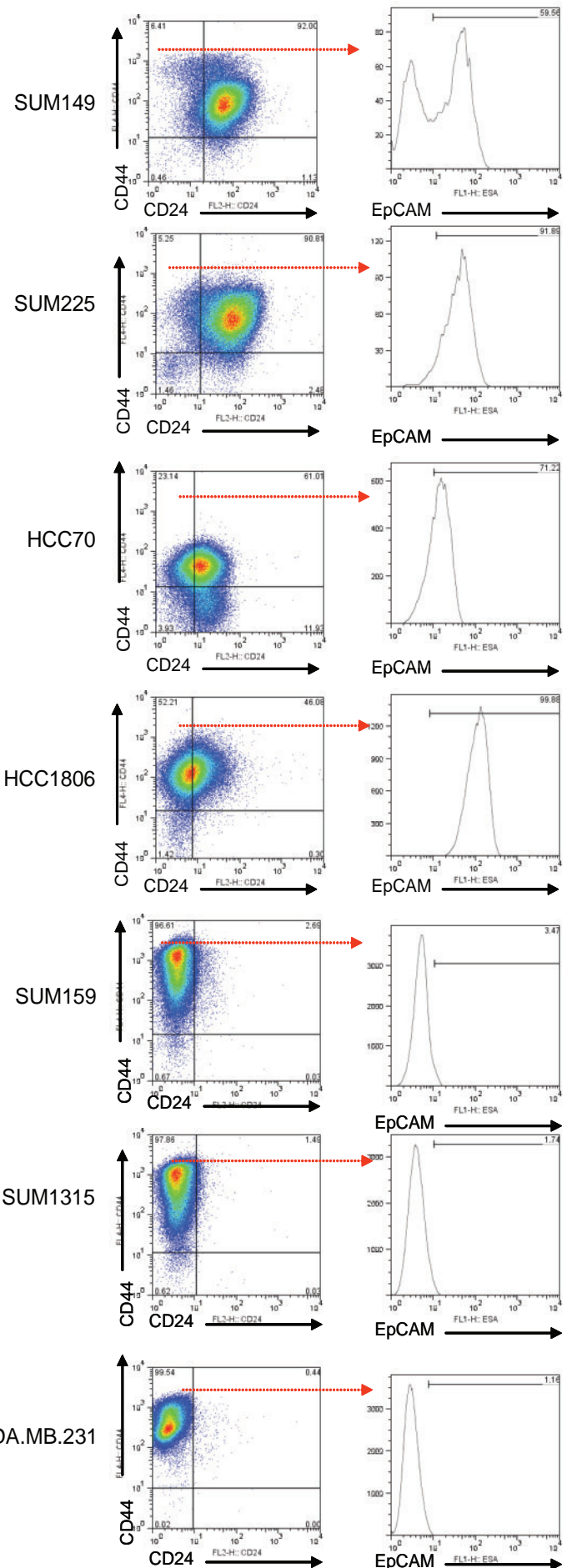

Supplement: Additional file 3 — CD44+/CD24-/EpCAM+ cells are variable across a panel of cultured human breast cell lines. Human breast cancer cell lines were stained for EpCAM, CD24, and CD44 and quantified by flow cytometry as described in Materials and Methods. Cell staining CD44+/CD24- (upper left quadrant, dot plots) were analyzed for the percentage of EpCAM+ cells, which is shown in the histogram to the right of the dot plots. The percentage of CD44+/CD24-/EpCAM+ cells is calculated by multiplying the percentage of EpCAM+ cells by the percentage of CD44+/CD24+ cells. [file bcr2755-S3.PDF]

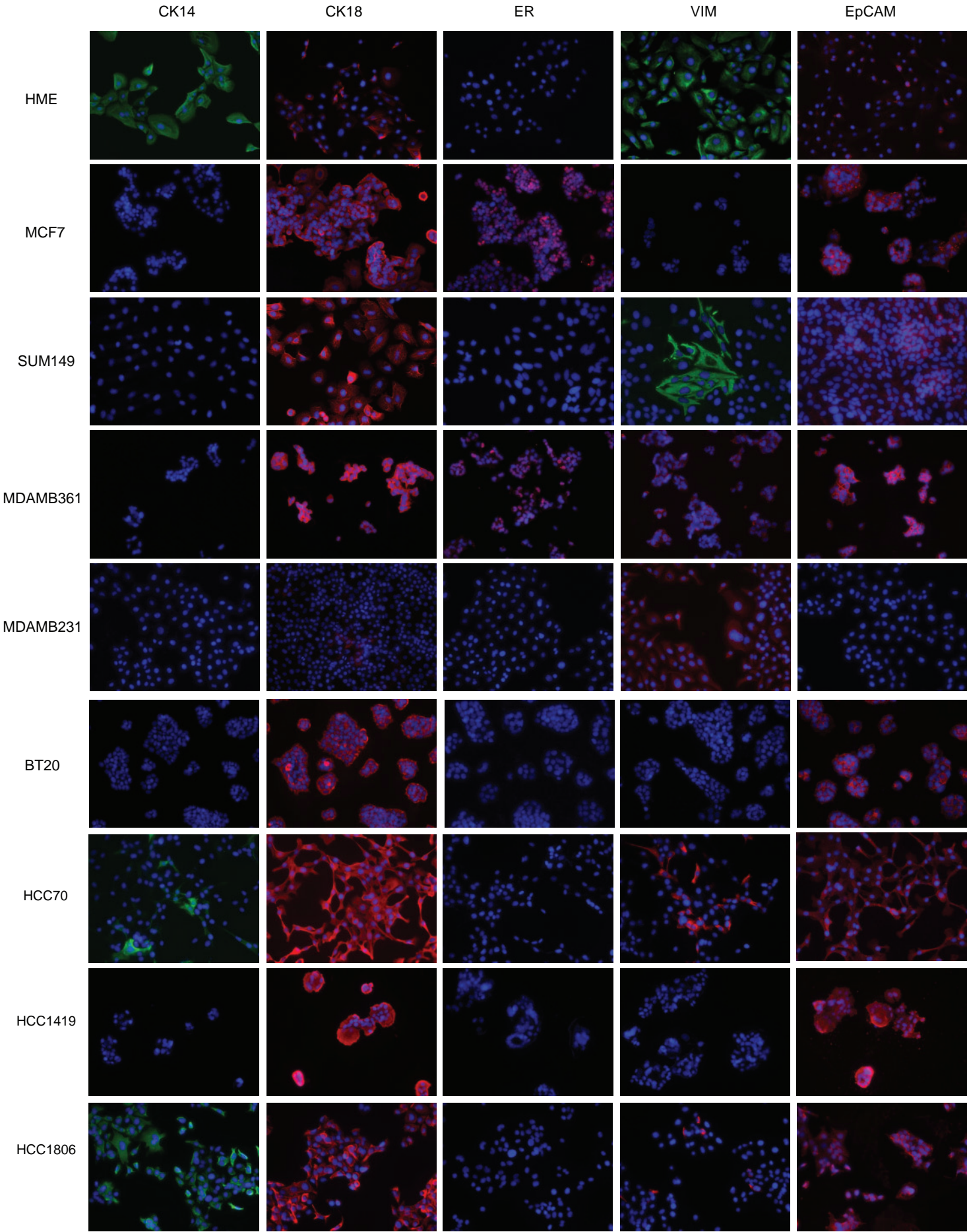

Supplement: Additional file 4 — Luminal 1, Luminal 2, Basal, and Mesenchymal cell lines identified by EpCAM, CD24, and CD49f expression were classified on the basis of CK14, CK8/18, ERα, EpCAM, and vimentin expression. Representative immunofluorescent images are from the panel of Luminal 1, Luminal 2, Basal, and Mesenchymal cell lines. Nuclei were counterstained with DAPI (blue). Original magnification: 200×. [file bcr2755-S4.PDF]
