# Supplementary material for: Mapping the cellular and molecular heterogeneity of normal and malignant breast tissues and cultured cell lines
Source: Breast Cancer Res. 2010 Oct 21;12(5):R87. doi: 10.1186/bcr2755 (PMC3096980; doi:10.1186/bcr2755)
Supplement: Additional file 5 — Table 1. Molecular and cellular characterization of human breast cell lines. [file bcr2755-S5.PDF]

Table 1: Molecular Characterization of Human Breast Cell Lines

Cell Line	Source	*%CD44+ CD24-	Cellular Morphology	Cell Surface Differentiation State	Marker Expression in vitro					Cell Profile Classification		
					CK14	*CK18*	*ER*	*VIM*	*EpCAM*		*(Neve et al)*	*(Hollestelle et al)*
**HME I**	Reduction Mammoplasty	0.05	epithelial	EpCAM+/CD24-/CD49f+	pos	mixed	neg	pos	pos	BAS	ND	ND
**HMEII**	Reduction Mammoplasty	0.82	epithelial	EpCAM-/CD49f+	pos	mixed	neg	pos	mixed	MES	ND	ND
**MCF10F**	fibrocyctic Disease	52.53	epithelial	EpCAM+/CD24-/CD49f+	ND	ND	ND	ND	ND	BAS	ND	ND
**MCF10A**	fibrocyctic Disease	0.03	epithelial	EpCAM-/CD49f+	pos	pos	neg	pos	neg	MES	BASAL-B	ND
**MCF7**	Pleural Effusion	0.35	epithelial	EpCAM+/CD49f-	neg	pos	pos	neg	pos	LUM1	LUM	LUM
**HCC1428**	Pleural Effusion	3.88	epithelial	EpCAM+/CD49f-	neg	pos	pos	neg	pos	LUM1	LUM	ND
**T47D**	Pleural Effusion	0.53	epithelial	EpCAM+/CD49f+	neg	pos	pos	neg	pos	LUM2	LUM	LUM
**HCC1500**	Primary BrCa	N/D	epithelial	EpCAM+/CD49f+	neg	pos	pos	neg	pos	LUM2	BASAL-B	BASAL
**MDA.MB.361**	Pleural Effusion	0.04	epithelial	EpCAM+/CD49f+	neg	pos	pos	neg	pos	LUM2	LUM	LUM
**HCC1419**	Primary BrCa	0.05	epithelial	EpCAM+/CD49f+	neg	pos	neg	neg	pos	LUM2	ND	ND
**SUM225**	Chest Wall recurrence	5.25	epithelial	EpCAM+/CD49f+	neg	pos	neg	neg	pos	LUM2	BASAL-A	BASAL
**BT20**	Primary BrCa	0.53	epithelial	EpCAM+/CD49f+	neg	pos	neg	neg	pos	LUM2	BASAL-A	BASAL
**SUM149**	Primary BrCa	6.41	mixed	EpCAM+/CD24-/CD49f+	pos	pos	neg	mixed	pos	BAS	BASAL-B	BASAL
**SUM229**	Pleural Effusion	76.11	spindle	EpCAM+/CD24-/CD49f+	ND	ND	ND	ND	ND	BAS	ND	N/D
**HCC70**	Primary BrCa	23.14	spindle	EpCAM+/CD24-/CD49f+	pos	pos	neg	mixed	pos	BAS	BASAL-A	BASAL
**HCC1806**	Primary BrCa	52.51	spindle	EpCAM+/CD24-/CD49f+	pos	pos	neg	mixed	pos	BAS	ND	ND
**TUM177**	Primary BrCa	17.24	spindle	EpCAM-/CD49f+	neg	neg	neg	pos	neg	MES	N/A	N/A
**SUM1315**	Xenotransplant of met.	97.86	spindle	EpCAM-/CD49f+	neg	neg	neg	pos	neg	MES	BASAL-B	BASAL-B
**SUM159**	Primary BrCa	96.61	spindle	EpCAM-/CD49f+	neg	neg	neg	pos	neg	MES	BASAL-B	BASAL
**MDA.MB.231**	Pleural Effusion	99.54	spindle	EpCAM-/CD49f+	neg	neg	neg	pos	neg	MES	BASAL-B	BASAL

*Averaged over 3-4 independent biologcal replicates
N/A: Not applicable

ND: Not determined
mixed: >10% but < 50% of the cells spindle

pos: >80% of the cells stain
mixed: >1% but < 50% of the cells stain
neg; 0 % of the cells stain

33	-24
10	-29
-38	
